# Correction to ‘Using energy to go downhill—a genoprotective role for ATPase activity in DNA topoisomerase II’

**DOI:** 10.1093/nar/gkad1255

**Published:** 2023-12-28

*Nucleic Acids Research*, 2023; gkad1157, https://doi.org/10.1093/nar/gkad1157

In the originally published version of this manuscript, References (44-46) in the final paragraph do not link to the correct papers, and the correct references are missing from the list. Three references have been added to the end of the reference list as #52-54, and the ‘(44-46)’ citation in the final paragraph has been replaced with ‘(52-54)’.

The references are:

52. Ju,B.-G., Lunyak,V. V, Perissi,V., Garcia-Bassets,I., Rose,D.W., Glass,C.K., and Rosenfeld,M.G. (2006) A topoisomerase IIbeta-mediated dsDNA break required for regulated transcription. *Science*, 312, 1798–1802.

53. Haffner,M.C., Aryee,M.J., Toubaji,A., Esopi,D.M., Albadine,R., Gurel,B., Isaacs,W.B., Bova,G.S., Liu,W., Xu,J., *et al.* (2010) Androgen-induced TOP2B-mediated double-strand breaks and prostate cancer gene rearrangements. *Nat. Genet*., 42, 668–675.

54. Trotter,K.W., King,H.A., and Archer,T.K. (2015) Glucocorticoid Receptor Transcriptional Activation via the BRG1-Dependent Recruitment of TOP2β and Ku70/86. *Mol. Cell. Biol*., 35, 2799–2817.

This error has been corrected online.

