# Supplementary material for: Using energy to go downhill—a genoprotective role for ATPase activity in DNA topoisomerase II
Source: Nucleic Acids Res. 2023 Dec 1;52(3):1313–24. doi: 10.1093/nar/gkad1157 (PMC10853770; doi:10.1093/nar/gkad1157)
Supplement: gkad1157_supplemental_file [file gkad1157_supplemental_file.pdf]

Table S1. Recombination frequency induced by expression of headless hTOP2β HL. CG2009, genotype MATa/MATa lys2-1/lys2-2 tyr1-1/tyr1-2 his7-2/his7-1 leu2D/leu2D ura3D/ura3-1 trp5-d/trp5-c met13-d/met13-c ade5/ADE5 ade2/ade2 was transformed with the indicated plasmids, and individual single colony transformants were inoculated in yeast synthetic complete media. After overnight incubation, cultures were plated to media lacking histidine (His-) lysine (Lys-) or tryptophan (Trp-) to select for heteroallelic recombination at HIS7, LYS2 or TRP5. Diluted cultures were also plated to media lacking uracil to determine the number of viable plasmid carrying cells. Recombination frequencies were calculated by dividing the number of colonies on the relevant plates by the total viable count. Recombination frequencies are expressed x10^4 and are shown mean ± SD from five independent transformants. pDEDyTop2 overexpresses wildtype yTOP2 from the DED1 promoter. pDED yTOP2 R1128G F1025Y expresses a self-poisoning TOP2 allele that leads to elevated recombination and is a positive control for the recombination induction(28).

**CG2009**	**His-**	**Lys-**	**Trp-**
Ycplac33	0.13 ± 0.030	0.17 ± 0.024	0.65 ± 0.11
pDED yTOP2	0.029 ± 0.0052	0.22 ± 0.032	0.42 ± 0.057
pDED yTOP2 R1128G F1025Y	0.52 ± 0.048	0.34 ± 0.028	1.5 ± 0.092
pKN17 hTOP2β wildtype full-length	0.25 ± 0.045	0.16 ± 0.008	0.57 ± 0.024
Headless hTOP2β HL	1.8 ± 0.19	2.3 ± 0.044	4.3 ± 0.20

Table S2. Primers used in this study.

Name	Primer
hTOP2β 12UraC K600T_F	CATTACTCCTATTGTAACGGCAAGCAAAAATAAGC
hTOP2β 12UraC K600T_R	GCTTATTTTTGCTTGCCGTTACAATAGGAGTAATG
hTOP2β 12UraC R757W_F	GTTTCAAGTGGAATGATAAACGTGA
hTOP2β 12UraC R757W_R	AGGTAAATAAAACTTTCCGCTGG
hTOP2β 2BT Y821F_F	GATGCTGCAAGCCCTCGTTTTATTTTCACAATGTTAAGC
hTOP2β 2BT Y821F_R	GCTTAACATTGTGAAAATAAAACGAGGGCTTGCAGCAT
hTOP2α 2BT Y805F_F	GATTCTGCTAGTCCACGATTCATCTTTACAATGCTCAGC
hTOP2α 2BT Y805F_R	GCTGAGCATTGTAAAGATGAATCGTGGACTAGCAGAATC
hTOP2β HL 5' truncation_F	AACACATGTGGATATCTTGACTGATTTTTCCATGGAGG
hTOP2β HL 5' truncation_R	TTACTGATGACATGGGGCTGCAGGAATTCCTG
hTOP2β HL 3' end_F	TTCCTGCAGCCCCATGTCATCAGTAAAATACAGTAAAATC
hTOP2β HL 3' end_R	AATCAGTCAAGATATCCACATGTGTTTTTAGTAAAC

Fig. S1

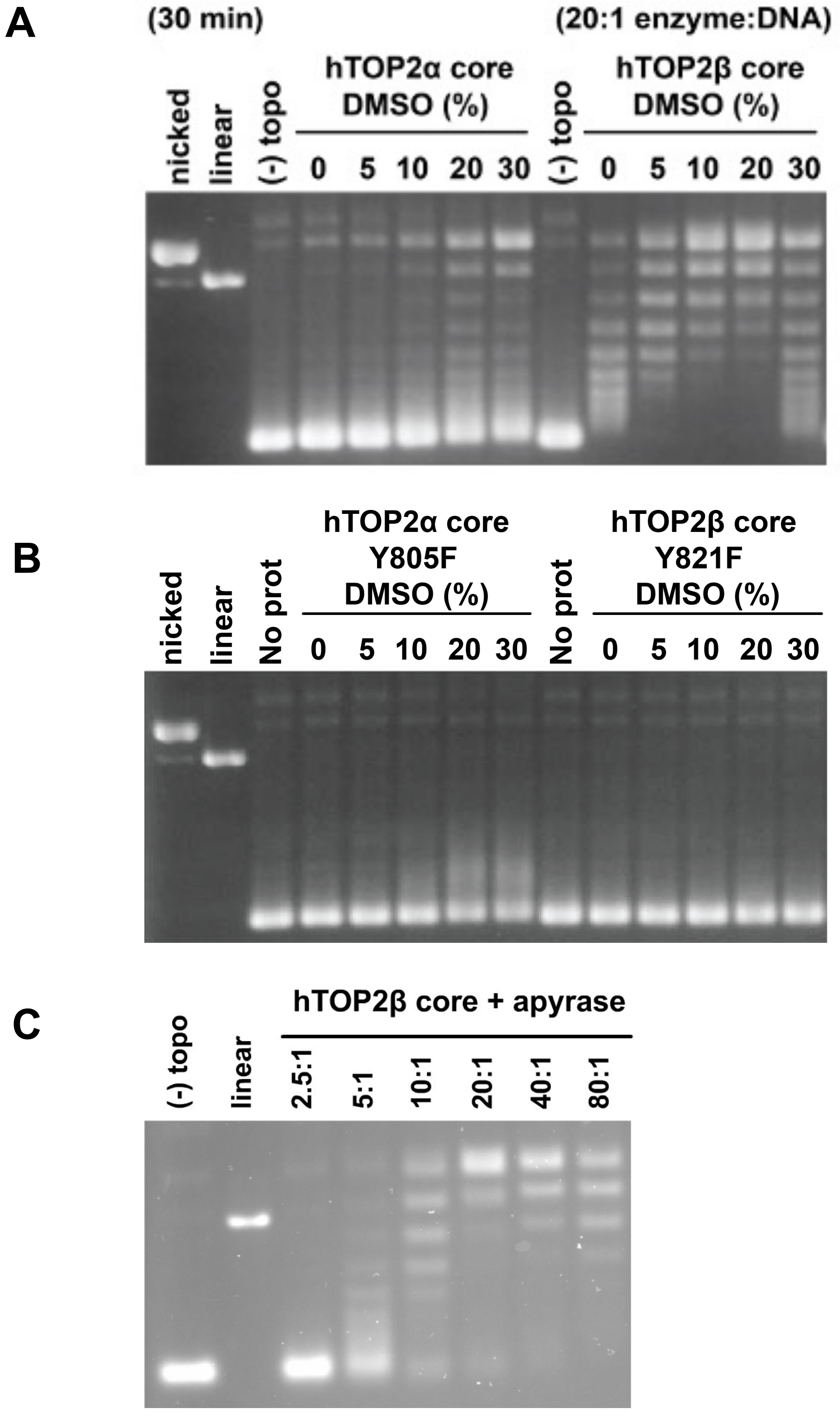

Fig. S1. DMSO enhances supercoil relaxation activity of the hTOP2α and hTOP2β cores. (A) Activity of the hTOP2α and hTOP2β cores on negatively supercoiled plasmid DNA as titrated against increasing concentrations of DMSO. (B) Substitution of the catalytic tyrosine in the hTOP2α and hTOP2β cores abolishes ATP-independent supercoil relaxation activity. (C) Treatment of reactions with apyrase does not impede supercoil relaxation by the hTOP2β core, further corroborating the ATP independence of the reaction.

Fig. S2

A

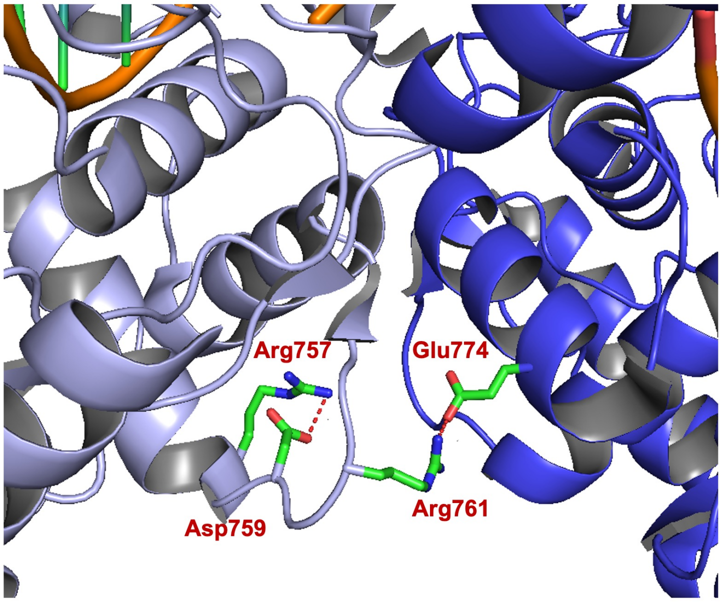

B

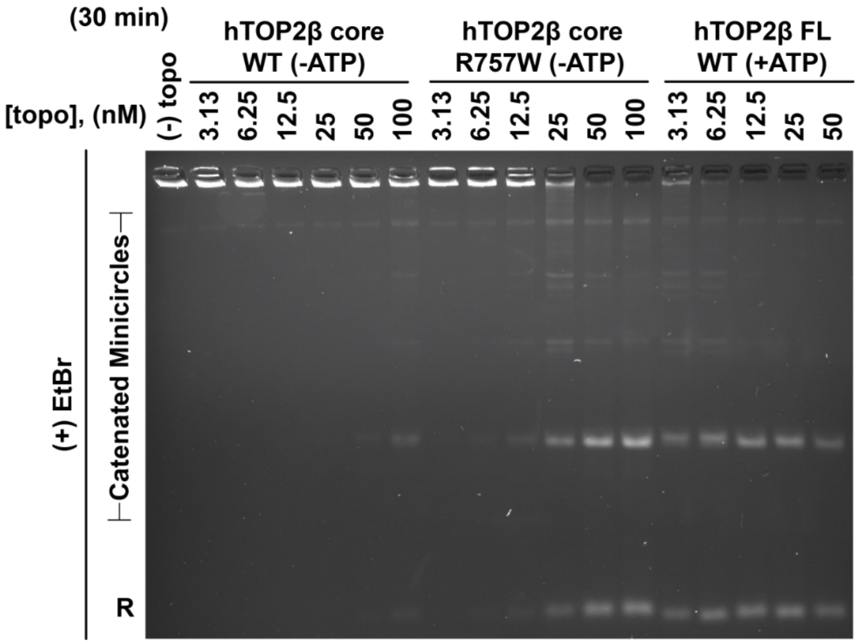

Fig. S2. The hTOP2β^{R757W} core has enhanced decatenation activity. (A) Location of Arg757 (PDB: 3QX3). (B) Decatenation of kDNA by the wildtype hTOP2β core, the hTOP2β^{R757W} core and wildtype, full-length hTOP2β. Increasing amounts of each enzyme were incubated with 250 ng of kDNA for 30 min at 37 ˚C, in the absence ((-), for the cores) or presence ((+), for full-length) of ATP.

Fig. S3

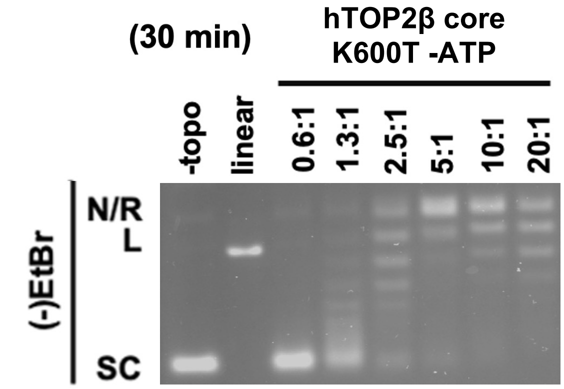

Fig. S3. The hTOP2β^{K600T} core has enhanced relaxation activity and cleavage propensity relative to the wildtype hTOP2β core. Ratios refer to enzyme dimers:DNA. Enzymes were incubated with 10 ng/μL of negatively supercoiled pSG483 for 30 min at 37 ˚C.

Fig. S4

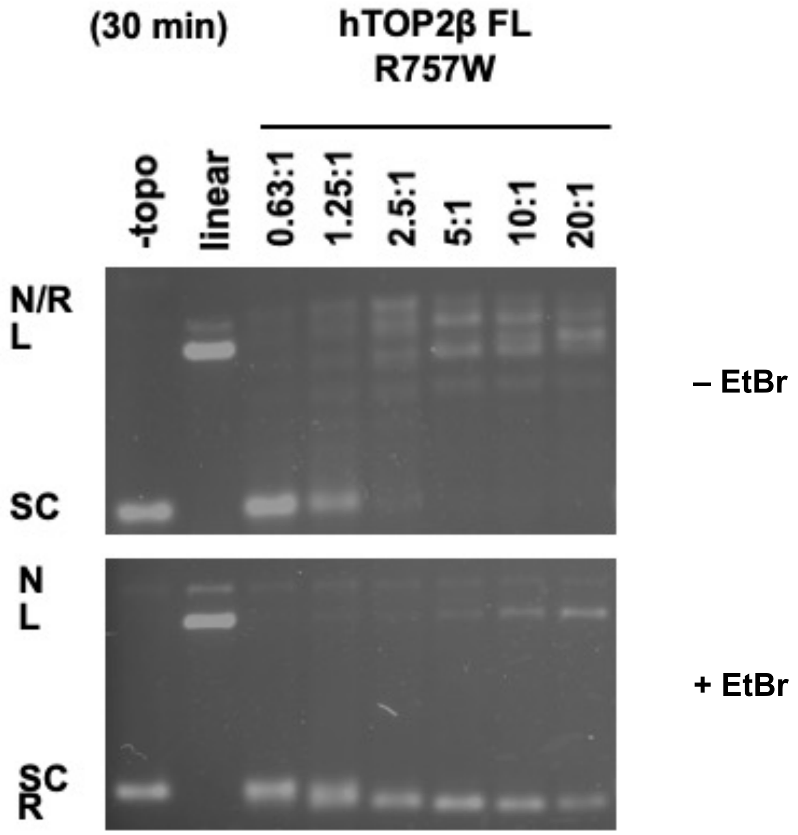

Fig. S4. Full-length hTOP2β^{R757W} has reduced cleavage activity compared to the hTOP2β^{R757W} core. Activity of full-length hTOP2β^{R757W} on negatively supercoiled plasmid DNA, separated in agarose gels in the absence (upper panel) or presence (lower panel) of ethidium bromide (EtBr). Compare with **Fig. 3**.

Fig. S5

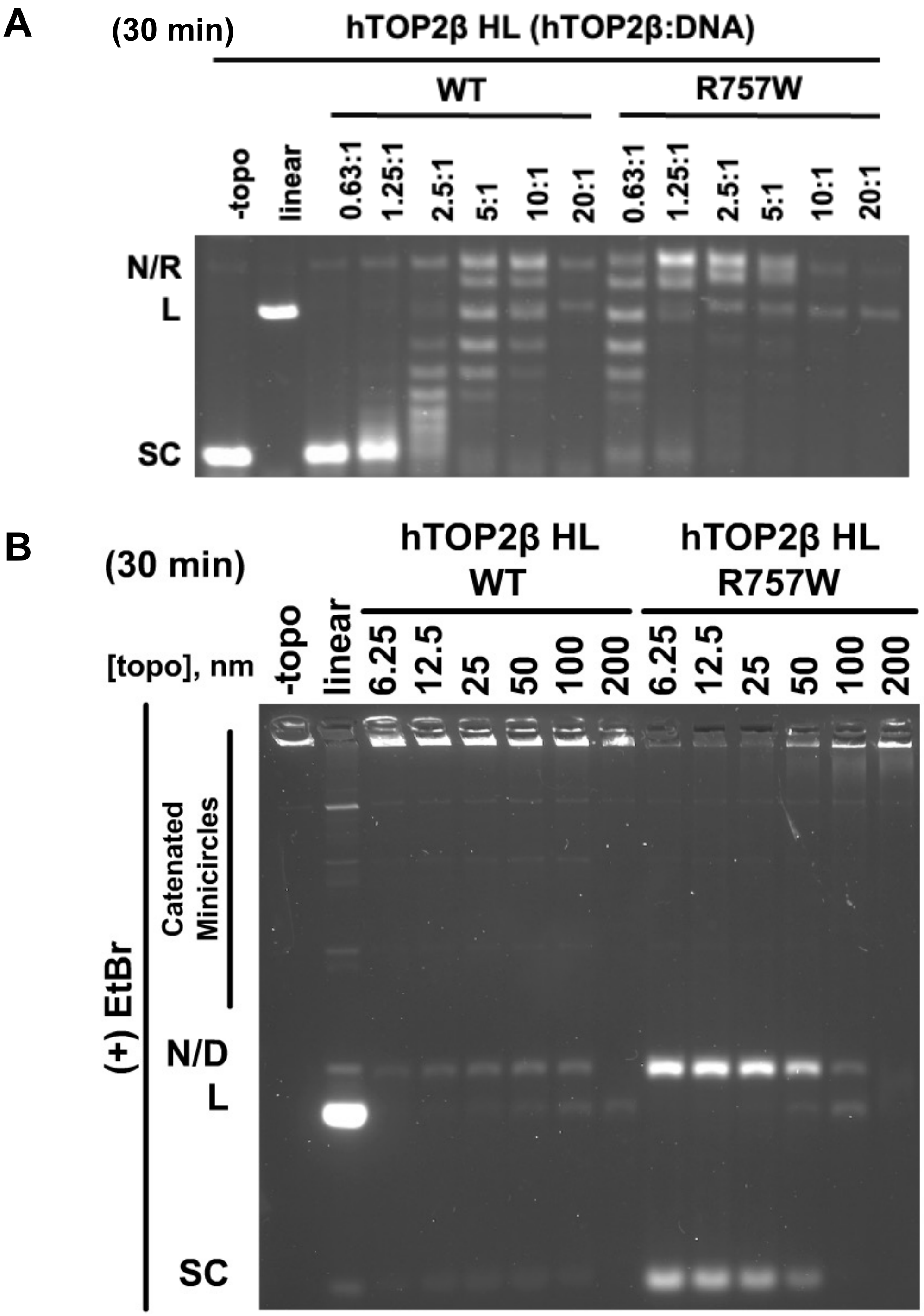

Fig. S5. Headless hTOP2β^{R757W} has enhanced relaxation and decatenation activities over wildtype headless hTOP2β. (A) Activity of headless wildtype hTOP2β and hTOP2β^{R757W} on negatively supercoiled plasmid DNA. Ratios refer to enzyme dimers:DNA. (B) Decatenation of kDNA by headless wildtype hTOP2β and hTOP2β^{R757W}. Increasing amounts of each enzyme were incubated with 250 ng of kDNA for 30 min at 37 ˚C.
